# Assessing and Resolving Model Misspecifications in Metabolic Flux Analysis

**DOI:** 10.3390/bioengineering4020048

**Published:** 2017-05-24

**Authors:** Rudiyanto Gunawan, Sandro Hutter

**Affiliations:** 1 Institute for Chemical and Bioengineering, Department of Chemistry and Applied Biosciences, ETH Zurich, 8093 Zurich, Switzerland; sandro.hutter@chem.ethz.ch; 2 Swiss Institute of Bioinformatics, 1015 Lausanne, Switzerland

**Keywords:** metabolic flux analysis, model misspecification, constraint-based model, stoichiometric model, Chinese hamster ovary cell culture

## Abstract

Metabolic flux analysis (MFA) is an indispensable tool in metabolic engineering. The simplest variant of MFA relies on an overdetermined stoichiometric model of the cell’s metabolism under the pseudo-steady state assumption to evaluate the intracellular flux distribution. Despite its long history, the issue of model error in overdetermined MFA, particularly misspecifications of the stoichiometric matrix, has not received much attention. We evaluated the performance of statistical tests from linear least square regressions, namely Ramsey’s Regression Equation Specification Error Test (RESET), the F-test, and the Lagrange multiplier test, in detecting model misspecifications in the overdetermined MFA, particularly missing reactions. We further proposed an iterative procedure using the F-test to correct such an issue. Using Chinese hamster ovary and random metabolic networks, we demonstrated that: (1) a statistically significant regression does not guarantee high accuracy of the flux estimates; (2) the removal of a reaction with a low flux magnitude can cause disproportionately large biases in the flux estimates; (3) the F-test could efficiently detect missing reactions; and (4) the proposed iterative procedure could robustly resolve the omission of reactions. Our work demonstrated that statistical analysis and tests could be used to systematically assess, detect, and resolve model misspecifications in the overdetermined MFA.

## 1. Introduction

The ability of biological systems to produce highly complex molecules at high enantiomeric excess has pushed metabolic engineering that relies on directed alterations of the cell’s biochemical reactions through recombinant DNA technology to the center stage of biotechnology [1,2]. The understanding of cellular metabolism and its manipulation encompass much of the research activities in modern biotechnology. Mathematical modeling of cellular metabolism, particularly constraint-based or stoichiometric modeling, has been playing an important role not only in the analysis of metabolic phenotypes (metabolic flux distribution) but also in the design and optimization of metabolic pathways to enhance productivity or to synthesize new desired products. The most widely used model-based analysis in metabolic engineering is the metabolic flux analysis (MFA), which comprises methods for determining intracellular metabolic fluxes. MFA employs a stoichiometric model of the metabolic reaction network based on the mole balance equation of the intracellular metabolites under a pseudo-steady state assumption [1,3]. 

A simple strategy of MFA, from here on referred to as the overdetermined MFA, uses a reduced stoichiometric model of the cell’s metabolism such that the estimation of the metabolic fluxes is mathematically well-posed, i.e., the flux estimation involves an (over)determined system. For larger and more realistic metabolic models, the flux estimation in the MFA often becomes underdetermined as the number of unknown fluxes exceeds the number of balance equations. There has been a flurry of activity in the development of MFA methods for large metabolic models based on linear programming (notably flux balance analysis) [4], which goes hand in hand with the creation of genome-scale metabolic models. While the MFA strategies above are predominantly based on measurements of extracellular concentration of metabolites, a different class of MFA techniques that rely on data from ^13^C isotopic labeling experiments has emerged and matured over the past two decades. The experimental, analytical, and computational procedures for ^13^C-based MFA have now been standardized [5].

Due to the experimental and computational complexity in the application of MFA using genome-scale metabolic models and ^13^C isotopic labeling experiments, the overdetermined MFA continues to be used in practice, thanks to its simple formulation and numerical implementation [6,7]. A common criticism of the overdetermined MFA is the use of a reduced (incomplete) description of the metabolic network. The accuracy of the flux estimates is often a concern in the application of the overdetermined MFA, and the formulation of an appropriate reduced order model for a given set of extracellular species measurements in a particular organism is challenging. Past studies have established several guidelines for good practices in the overdetermined MFA. For example, analytical conditions that guarantee the ability of the stoichiometric model to balance, i.e., the measured rates of extracellular species concentrations can be balanced, and ensure the existence of a unique solution for the intracellular metabolic fluxes have been formulated [8]. In consideration of data noise (measurement errors), statistical tests on the goodness of fit could be used to assess the consistency between the data and the stoichiometric model [9,10]. The accuracy of the flux estimates could also be quantified by computing the corresponding confidence intervals [11] or by propagating known errors in the measurements to the flux estimates [12]. Finally, the significance of observed changes in the flux distribution between conditions or strains could and should be established by standard statistical tests (e.g., *t*-test) [13].

The test for goodness of fit or statistical significance of regression in the overdetermined MFA could fail because of several reasons, including (1) incorrect assumptions abuot the characteristics of data noise (e.g., on the mean and variance of the noise) and (2) model specification errors [14,15]. In such a scenario, the resulting flux estimates of the overdetermined MFA may have large inaccuracy or bias. Procedures for detecting and locating gross measurement errors and missing or incorrectly specified components have previously been proposed based on either the improvement of goodness of fit upon the removal of a measured variable [9] or the directionality of the residual vectors [14]. Despite its long history, the assessment, detection, and rectification of misspecifications of the stoichiometry matrix in the overdetermined MFA have not received much attention. In a recent study, Sokolenko et al. provided a procedure for detecting model error through the in silico generation of flux profiles and the common statistical *t*-tests [16]. 

In this work, we adapted statistical analysis and tools for model misspecifications commonly used in the field of linear least square regression to address the issue of missing reactions in the overdetermined MFA. We posited that the simplification of the stoichiometric model, either manually or using a numerical algorithm [17], to generate an overdetermined flux estimation problem in the overdetermined MFA may inadvertently remove important metabolic reactions. In this study, we illustrated how an omission of reaction(s) could lead to biases in the flux estimates, and evaluated the performance of several statistical tests including Ramsey’s RESET test, the F-test, and the Lagrange multiplier test, to detect such specification errors. Finally, we proposed an iterative procedure based on the F-test to resolve the issue of missing reactions. We demonstrated the ability of the aforementioned model misspecification tests and correction procedure by applying them to the flux analysis of Chinese hamster ovary metabolism and in silico metabolic networks.

## 2. Materials and Methods

### 2.1. Metabolic Flux Analysis

The MFA is based on the mole balance equation for the intracellular metabolites under a pseudo steady state assumption, as follows:

(1) $$\frac{dc}{dt}=Sv=0$$

where **c** is the vector of *m* metabolite concentrations, **v** denotes for the vector of *n* metabolic fluxes, and **S** denotes the m×n stoichiometric matrix. The fluxes describe either the rate of reactions that consume or produce the metabolites or the rate of the transport of metabolites between the cell and the extracellular environment or between different intracellular compartments. In the typical formulation of MFA, some, if not all, of the exchange fluxes, i.e., the fluxes of metabolites in and out of the cell, could be estimated from the measurements of extracellular species concentrations. The task at hand is to estimate the unknown internal metabolic fluxes from the exchange fluxes. For such a purpose, we begin with partitioning the stoichiometric matrix **S** and the flux vector **v** into:

(2) $$Sv=\left[\begin{array}{cc} S_{E} & S_{I} \end{array}\right]\left[\begin{array}{c} v_{E}\\ v_{I} \end{array}\right]=S_{E}v_{E}+S_{I}v_{I}=0$$

where the subscripts E and I refer to the exchange and internal fluxes, respectively. Thus, given the values of exchange fluxes $v_{E}$, the estimation of unknown internal fluxes $v_{I}$ is reduced to solve the following linear equation:

(3) $$S_{I}v_{I}=-S_{E}v_{E}$$

The estimation of the internal metabolic fluxes $v_{I}$ as stated in Equation (3) could be cast as a linear least square regression problem:

(4) y=Xβ+e

where **y** denotes the vector of measured response variables, **X** denotes the (non-random) design matrix containing the values of explanatory variables, β denotes the unknown parameter vector, and e denotes the vector of measurement errors (noise). The ordinary least square (OLS) estimate of the parameter β is given by the minimum of the following error function:

(5) $$\Phi_{\mathrm{OLS}}\left(\beta \right)={\left(y-X\beta \right)}^{T}\left(y-X\beta \right)$$

By invoking the first order necessary condition for optimality ($d\Phi_{\mathrm{OLS}}/db=0$), the OLS parameter estimate is given by:

(6) $${\hat{\beta }}_{\mathrm{OLS}}={\left(X^{T}X\right)}^{-1}X^{T}y$$

According to the Gauss-Markov theorem [18], when the measurement errors are additive and uncorrelated with zero mean and constant variance (i.e., $\mathrm{Cov}\left(e\right)=\sigma^{2}I$), ${\hat{\beta }}_{\mathrm{OLS}}$ gives the minimum variance unbiased estimate (MVUE) of β among all linear estimators, with the following variance-covariance matrix:

(7) $$\mathrm{Cov}\left({\hat{\beta }}_{\mathrm{OLS}}\right)={\left(X^{T}X\right)}^{-1}X^{T}\mathrm{Cov}\left(e\right)X{\left(X^{T}X\right)}^{-1}=\sigma^{2}{\left(X^{T}X\right)}^{-1}$$

If the measurement errors are correlated and/or have unequal variance with a known variance-covariance matrix Cov(e), then one could resort to the generalized least square (GLS) formulation by minimizing the following error function:

(8) $$\Phi_{\mathrm{GLS}}\left(\beta \right)={\left(y-X\beta \right)}^{T}\mathrm{Cov}{\left(e\right)}^{-1}\;\left(y-X\beta \right)$$

Naturally, the GLS estimation requires the variance-covariance matrix Cov(e) to be invertible. When $\mathrm{Cov}\left(e\right)=LL^{T}$ is invertible, the GLS is equivalent to the OLS regression for the linear problem $L^{-1}y=L^{-1}X\beta +L^{-1}e$. The GLS parameter estimate ${\hat{\beta }}_{\mathrm{GLS}}$ is therefore given by:

(9) $${\hat{\beta }}_{\mathrm{GLS}}={\left(X^{T}\mathrm{Cov}{\left(e\right)}^{-1}X\right)}^{-1}X^{T}\mathrm{Cov}{\left(e\right)}^{-1}y$$

Here, ${\hat{\beta }}_{\mathrm{GLS}}$ is the MVUE of β with the following variance-covariance matrix:

(10) $$\mathrm{Cov}\left({\hat{\beta }}_{\mathrm{GLS}}\right)={\left(X^{T}\mathrm{Cov}{\left(e\right)}^{-1}X\right)}^{-1}$$

By drawing parallels between the linear equation of the MFA in Equation (3) and the least square regression problem in Equation (4), we could set $y=-S_{E}v_{E}$, $X=S_{I}$, and $\beta =v_{I}$ and use the OLS or GLS formulation, whichever is appropriate, to obtain the flux estimate ${\hat{v}}_{I}$. Note that the existence of an optimal parameter estimate for the OLS (or GLS) requires the matrix $X^{T}X$ $\left(\mathrm{or}\;X^{T}\mathrm{Cov}{\left(e\right)}^{-1}X\right)$ to be invertible, i.e., the matrix **X** needs to have a full column rank. In the context of the overdetermined MFA, the rank condition puts a constraint on the dimension of the matrix $S_{I}$, such that the number of unknown internal fluxes $v_{I}$. should not exceed the number of metabolite species in the mole balance equation. The rank condition also necessitates that the reactions be linearly independent, i.e., each of the reactions could not be written as a linear combination of the other reactions. Upon violation of the above rank condition, for example when the number of reactions exceeds that of the species, the non-zero degrees of freedom in the mole balance equation allow the existence of many solutions (not all physically or biologically feasible), a problem that is addressed by strategies such as the flux balance analysis [4]. In this work, we focused on the MFA, with the matrix $S_{I}$. having a full column rank. 

### 2.2. Model Misspecification

Due to the rank condition on $S_{I}$, one often faces the challenge of choosing a small number of reactions, for example from the genome scale metabolic models, to include in the mole balance equation used in the overdetermined MFA. Despite the long history of MFA, the impact of an incorrect stoichiometric matrix specification, particularly due to the omission of reactions, on the accuracy of the flux estimate has not received much attention. Recently, Sokolenko et al. used the GLS framework, statistical *t*-test, and simulated flux values to show that model errors could lead to gross deviations in flux estimates that are not statistically significant [16]. In the field of linear least square regression, the impact of a model misspecification on the parameter estimates is well studied, and several tests have previously been developed to detect model misspecifications. In this study, we evaluated the performance of several such tests, including Ramsey’s RESET test, the F-test, and the Lagrange-Multiplier test, in detecting specification errors of the stoichiometric matrix in the overdetermined MFA.

#### 2.2.1. Effects of Missing Reactions

In the following, we considered the problem of missing or omitted reactions in the stoichiometric matrix. We assume that the metabolic network model in the MFA given in Equation (1) is incomplete and that the true mole balance is governed by:

(11) $$\;\left[\begin{array}{ccc} S_{E} & S_{I} & S_{O} \end{array}\right]\left[\begin{array}{c} v_{E}\\ v_{I}\\ v_{O} \end{array}\right]=S_{E}v_{E}+S_{I}v_{I}+S_{O}v_{O}=0$$

where $S_{O}$ contains the stoichiometric coefficients of the omitted reactions and $v_{O}$ denotes the vector of the metabolic fluxes of the omitted reactions. The least square problem of estimating the unknown metabolic fluxes $v_{I}$ and $v_{O}$ from the measurements of the exchange fluxes $v_{E}$ is given by:

(12) $$-S_{E}v_{E}=S_{I}v_{I}+S_{O}v_{O}+u$$

where **u** denotes the vector of measurement errors for the true model. Without a loss of generality, in order to illustrate the impact of omitting reactions, we could consider the OLS estimate of the internal fluxes $v_{I}$ using the mole balance in Equation (2), as follows:

(13) $${\hat{v}}_{I}={\left({S_{I}}^{T}S_{I}\right)}^{-1}{S_{I}}^{T}\left(-S_{E}v_{E}\right)$$

Substituting $-S_{E}v_{E}$ from the true model in Equation (12) into the OLS estimate from the misspecified model as stated in Equation (13) above, we obtain:

(14) $${\hat{v}}_{I}={\left({S_{I}}^{T}S_{I}\right)}^{-1}{S_{I}}^{T}\left(S_{I}v_{I}+S_{O}v_{O}+u\right)\;{\hat{v}}_{I}=v_{I}+{\left({S_{I}}^{T}S_{I}\right)}^{-1}{S_{I}}^{T}S_{O}v_{O}+{\left({S_{I}}^{T}S_{I}\right)}^{-1}{S_{I}}^{T}u$$

The above formula could also be derived using the GLS estimation whenever appropriate. Therefore, even when the measurement error **u** has zero mean, the flux estimate ${\hat{v}}_{I}$ may no longer be unbiased, i.e., the expected value of ${\hat{v}}_{I}$ may not equal to $v_{I}$. More specifically, the specification bias caused by the missing reactions is given by:

(15) $$\mathrm{bias}=E\left({\hat{v}}_{I}\right)-v_{I}={\left({S_{I}}^{T}S_{I}\right)}^{-1}{S_{I}}^{T}S_{O}v_{O}$$

where $E\left({\hat{v}}_{I}\right)$ is the expected value of ${\hat{v}}_{I}$. Therefore, the specification bias scales with the degree of correlations between the stoichiometry of the accounted reactions in $S_{I}$ and that of the omitted reactions in $S_{O}$ (from ${S_{I}}^{T}S_{O}$) and with the magnitude of the omitted reaction fluxes $v_{O}$. As illustrated in Case Study I below, the omission of a single reaction could lead to a large bias in the flux estimate. 

The derivation above shows how the omission of or failure to include reactions in the stoichiometric model could cause a bias in the flux estimate in the overdetermined MFA. In addition to the flux bias, missing reactions would also result in a lower variance, i.e., the variance of the OLS estimate of $v_{I}$ computed using the (misspecified) model in Equation (2) is smaller than the variance of the OLS estimate computed using the (true) model in Equation (11) [19]. Under certain conditions, the mean square error of the OLS estimate of the misspecified model may also be lower than that of the true model [19]. On the contrary, the inclusion of irrelevant reactions in the stoichiometry matrix, wherein **S** contains reactions that are non-existent in the actual system, would not introduce any bias to the OLS estimate of the fluxes. However, having additional reactions would artificially increase the variance and mean square error of the flux estimate [19].

#### 2.2.2. Model Misspecification Tests 

There exist several tests to detect model misspecification in a linear least square regression. In this study, we focus on tests of the misspecification of regression mean; whether **Xβ** is a good description of the response variable **y**. While tests for checking the validity of constant variance or the normality of the error variables in the least square regression also exist, we do not deal with these so-called misspecifications of higher moments. Interested readers are referred to the article by Long and Trivedi [20]. The first misspecification test under evaluation is the Ramsey’s RESET (Regression Equation Specification Error Test) test [21], which does not require any information on the possible missing variables (reactions) to formulate the hypothesis test. In the context of the overdetermined MFA, this scenario corresponds to when the stoichiometry of the missing reactions is unknown. The *p* order RESET test is based on the following linear least square problem:

(16) $$y=X\beta +\alpha_{1}{\hat{y}}^{2}+\ldots +\alpha_{p}{\hat{y}}^{p+1}+e^{*}$$

where $\hat{y}=\;X{\hat{\beta }}_{\mathrm{OLS}}$ and ${\hat{y}}^{p}$ are the vectors of *p* powers of ${\hat{y}}_{i}$ elements. The premise of the RESET test is that the contribution from the missing variables could be approximated by the powers of $\hat{y}$ (by way of a Taylor series expansion). The null hypothesis in the RESET test is that the coefficients $\alpha_{i}$’s are zero, i.e., H_0_: $\alpha_{1}=\ldots =\alpha_{p}=0$. The null hypothesis will be rejected if the following condition on the test statistic $S_{RESET}$ is satisfied:

(17) $$S_{RESET}=\frac{\left(\Phi_{\mathrm{OLS}}\left({\hat{\beta }}_{\mathrm{OLS}}\right)-\Phi_{\mathrm{OLS}}\left({\tilde{\beta }}_{\mathrm{OLS}},{\tilde{\alpha }}_{\mathrm{OLS}}\right)\right)/p}{\Phi_{\mathrm{OLS}}\left({\tilde{\beta }}_{\mathrm{OLS}},{\tilde{\alpha }}_{\mathrm{OLS}}\right)/\left(N-K-p\right)}>F_{p,N-K-p}\left(a\right)$$

where ${\hat{\beta }}_{\mathrm{OLS}}$ is given in Equation (6), ${\tilde{\beta }}_{\mathrm{OLS}}$ and ${\tilde{\alpha }}_{\mathrm{OLS}}$ are the OLS estimates for β and α for the regression problem in Equation (16), N is the length of the response vector **y**, *K* is the dimension of the unknown parameter β, and $F_{p,N-K-p}\left(a\right)$ is the (1−a)th percentile point of the Snedecor’s F distribution with *p* and N−K−p degrees of freedom. In essence, the inequality in Equation (17) is fulfilled when the additional parameters α in Equation (16) lead to a (statistically) significantly better data fitting than the linear equation in Equation (4). A rejection of the null hypothesis is therefore taken as strong evidence supporting the existence of a model functional misspecification. 

The next two misspecification tests apply to the scenario in which the stoichiometry of the candidate missing reactions are known. Given the current extensive knowledge on metabolic reactions, including the complete maps of metabolic networks for many major organisms and the availability of extensive and curated biochemical reaction databases (e.g., Kyoto Encyclopedia of Genes and Genomes (KEGG) [22], MetaCyc [23], Rhea [24]), one could efficaciously put together a list of possible missing reactions in the overdetermined MFA. Here, we consider the least square regression problem:

(18) $$y=X\beta +Z\alpha \;+\;e^{*}$$

where **Z** denotes the design matrix of the candidate missing variables; namely, the stoichiometric matrix of the possible missing reactions in the overdetermined MFA. 

The first test in this category is based on a similar null hypothesis as the Ramsey RESET test. The null hypothesis H_0_: α=0 will be rejected if the following condition of the test statistic $S_{F-test}$ is fulfilled: [25]

(19) $$S_{F-test}=\frac{\left(\Phi_{\mathrm{OLS}}\left({\hat{\beta }}_{\mathrm{OLS}}\right)-\Phi_{\mathrm{OLS}}\left({\bar{\beta }}_{\mathrm{OLS}},{\bar{\alpha }}_{\mathrm{OLS}}\right)\right)/o}{\Phi_{\mathrm{OLS}}\left({\bar{\beta }}_{\mathrm{OLS}},{\bar{\alpha }}_{\mathrm{OLS}}\right)/\left(N-K-o\right)}\geq F_{o,N-K-o}\left(a\right)$$

where ${\bar{\beta }}_{\mathrm{OLS}}$ and ${\bar{\alpha }}_{\mathrm{OLS}}$ are the OLS estimate for β and α for the regression problem in Equation (18) and *o* is the number of possible missing variables (i.e., the dimension of α). We refer the model misspecification test above as, for the lack of a better word, the F-test. Note that the existence of a unique solution of ${\bar{\beta }}_{\mathrm{OLS}}$ and ${\bar{\alpha }}_{\mathrm{OLS}}$ requires that the combined matrix $\left[\begin{array}{cc} X & Z \end{array}\right]$ has a full column rank. 

The third and last misspecification test in this work is derived from the Lagrange multiplier (LM) test, as proposed by Davidson and Mackinnon [26]. The premise of the LM test is to examine whether there exists a significant correlation between the residuals of the proposed linear model in Equation (4) and the part of the matrix **Z** in Equation (18), denoted by denoted by $Z^{c}$, which remains after removing the linear influence by **X**. In addition, the LM test also incorporates a heteroscedasticity-consistent (HC) covariance matrix to accommodate the situation when the variance of the measurement error is not constant. Following the procedure formulated in Long and Trivedi [20], we first compute the $Z^{c}$ as follows:

(20) $$Z^{c}=Z-\overset{˘}{X}{\left({\overset{˘}{X}}^{T}\overset{˘}{X}\right)}^{-1}{\overset{˘}{X}}^{T}Z$$

where $\overset{˘}{X}=\left[\begin{array}{cc} 1 & X \end{array}\right]$ and **1** is the (column) vector of 1s with the same row dimension as the matrix **X**. The test statistic is given by:

(21) $$S_{LM}={\hat{e}}^{T}Z^{c}{\left(\frac{N-K}{N-K-o}Z^{c^{T}}\hat{\Omega }Z^{c}\right)}^{-1}Z^{c^{T}}\hat{e}$$

where $\hat{e}=y-X{\hat{\beta }}_{\mathrm{OLS}}$ denotes the OLS residuals and $\hat{\Omega }$ is a diagonal matrix with the squares of the standard errors as the diagonal elements (${\hat{\Omega }}_{i,i}={\hat{e}}_{i}^{2}$). The null hypothesis H_0_: $Z^{c^{T}}\hat{e}=0$ is rejected if $S_{LM}\geq \chi_{o}^{2}\left(a\right)$, where $\chi_{o}^{2}\left(a\right)$ denotes the (1−a)th percentile point of the chi-square distribution with *o* degrees of freedom. The rejection of the null hypothesis due to a (statistically) significant correlation between the residual $\hat{e}$ and $Z^{c}$ is taken as strong evidence for the existence of a model misspecification. 

#### 2.2.3. Resolving Model Misspecification 

The rejection of the null hypothesis in the tests above does not immediately point to the identity of the missing or omitted reactions. Given the set of possible missing reactions, we could nevertheless apply the F-test or LM test to determine whether the addition of a reaction or a set of reactions among the possible missing reactions would significantly improve the linear regression. Reactions that are positively identified by the tests could therefore be added into the stoichiometric matrix. The procedure above can be repeated until no more reactions can be added. More precisely, we propose the following iterative procedure for correcting misspecifications in the stoichiometric matrix for the overdetermined MFA using the F-test:

1. Given the exchange fluxes **v_E_**, the stoichiometric matrices **S_E_** and **S_I_**, and the possible missing reaction stoichiometric matrix **S_A_**, we formulate the linear least square regression problem with $y=-S_{E}v_{E}$, $X=S_{I}$, and $\beta =v_{I}$.
2. Compute $S_{F-test}$ using **Z** constructed from every *k-*tuple combination of the columns (reactions) of **S_A_**.
3. Identify the *k-*tuple combination(s) satisfying $S_{F-test}\geq F_{o,N-K-o}\left(a\right)$ and move the corresponding columns from **S_A_** to **S_I_**.
4. Repeat steps 2 to 3 until no more reactions can be moved from **S_A_** to **S_I_**, that is, until the remaining set of *k*-tuple reaction combinations satisfying $S_{F-test}\geq F_{o,N-K-o}\left(a\right)$ is empty.

The procedure above is written generally for any *k-*tuple combination of reactions. In the case study, we performed the procedure, first using *k* = 1 (single reactions) and using *k* = 1 followed by *k* = 2 (pairs of reactions). If desired, the LM test could also be used in place of the F-test.

### 2.3. In Silico Metabolic Network Models and Data Generation

#### 2.3.1. Chinese Hamster Ovary Model 

**Figure 1:**
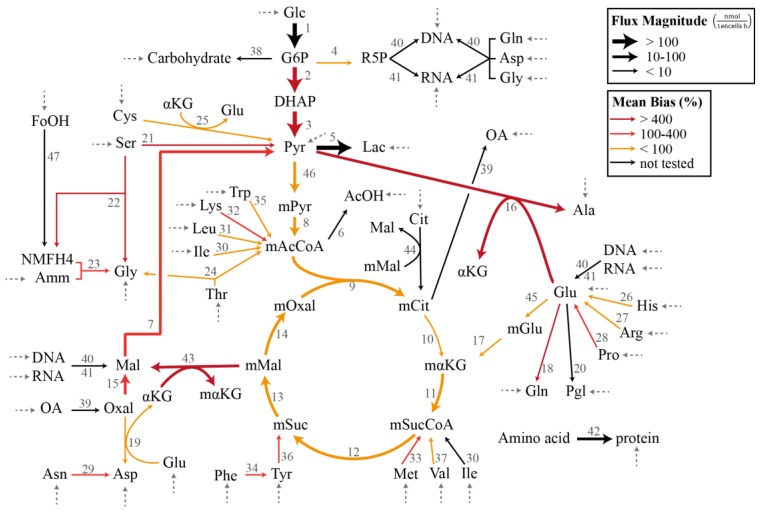
Chinese hamster ovary metabolic network model in Case Study I and III (adapted from [16,27]). The dashed arrows indicate the measured exchange (uptake) fluxes. The magnitudes of the flux estimates ${\hat{v}}_{I,GLS}$ are indicated by the thickness of the arrows, while the colors of the arrows represent the average relative magnitude of the specification bias caused by the removal of the reaction.

In the demonstration of the specification bias caused by missing reactions and the iterative procedure for resolving the stoichiometric matrix misspecification, we employed a metabolic network model previously created for the flux analysis of Chinese hamster ovary (CHO) batch cultivation data [16,27]. The model describes the concentration of 49 metabolites, out of which 34 are transported in and out of the cell by exchange fluxes. The stoichiometric matrix **S_I_** has a dimension of 49 metabolites and 47 internal fluxes with a full column rank. Figure 1 illustrates the CHO metabolic network model that corresponds to the stoichiometric matrix **S_I_** in the MFA. The complete list of reactions, the measured uptake flux values, and the standard deviations reported by Sokolenko et al. [16] are given in Supplementary Table S1. The standard deviation of the fluxes has a linear relationship with the uptake rates, as shown in Supplementary Figure S1.

#### 2.3.2. Random Metabolic Models

For a large-scale evaluation of the model misspecification tests, we generated in silico data $y=-S_{E}v_{E}$ using randomly generated stoichiometric matrices **S_I_** of various sizes (*m* = 50 to 200 metabolites and *n* = 55 to 220 reactions) and with different numbers of exchange fluxes (between 25 and 100). More precisely, for each data vector **y**, we employed the RMBNToolbox (MATLAB) [28] to generate a random stoichiometric matrix **S_I_** with the desired dimensions and a full column rank, wherein each species participated in at least one reaction. For the specified number of exchange fluxes *m_E_*, we assigned the first *m_E_* metabolites (rows) of **S_I_** as the species whose exchange fluxes were measured, while the remaining species only existed intracellularly (without exchange fluxes). More specifically, we used the matrix $S_{E}$ with the block structure $S_{E}=\left[\begin{array}{c} I_{m_{E}}\\ 0 \end{array}\right]$, where $I_{m_{E}}$ denotes the $m_{E}\times m_{E}$ identity matrix and 0 denotes the $\left(m-m_{E}\right)\times m_{E}$ zero matrix. Accordingly, we partitioned the matrix **S_I_** into:

(22) $$S_{I}v_{I}=\left[\begin{array}{c} S_{I,E}\\ S_{I,NE} \end{array}\right]v_{I}=-S_{E}v_{E}=\left[\begin{array}{c} -v_{E}\\ 0 \end{array}\right]$$

where $S_{I,E}$ corresponds to the first *m_E_* rows of the measured exchange fluxes and $S_{I,NE}$ corresponds to the remaining rows. We simulated the measured flux data in two steps: (1) we generated an internal flux vector **v_I_** using a linear combination of the kernel of $S_{I,NE}$ with uniform random coefficients between −1 and 1 and (2) we calculated the exchange fluxes using **v_I_** in Step (1) according to Equation (22) and contaminated the exchange flux vector with a vector of independent realizations of Gaussian distributed random numbers with zero mean and for different coefficients of variation (CoV of 1 to 20%). Note that since the noise standard deviation scales with the noise-free **y** (motivated by Supplementary Figure S1), only the first *m_E_* elements of **y** were non-zero. The misspecification tests were applied to the in silico generated data y, using the design matrix **X**, constructed by removing reactions randomly (between two to 20 reactions) from the matrix $S_{I}$. 

The performance of each misspecification test was judged based on the rate of true positive (TP), true negative (TN), false positive (FP), and false negative (FN) results. For the Ramsey RESET test, the true positives and false negatives were determined by applying the test to the generated data using the stoichiometric matrix $S_{I}$ with some of the reactions (columns) randomly removed. In this scenario, a rejection of the null hypothesis corresponded to a TP, while a non-rejection was a FN error. Meanwhile, the numbers of FP and TN of the RESET test were computed based on rejections and non-rejections of the null hypothesis, respectively, when applying the test using the full stoichiometric matrix $S_{I}$ (no missing reactions). The data generation and the RESET test were repeated 1000 times, each using a different randomly generated stoichiometric matrix $S_{I}$. 

In order to determine the TP and FN rates of the F-test and LM test, we generated in silico data **y** as described above and applied the tests using the stoichiometric matrix $S_{I}$ with a set of its reactions randomly removed. Here, the matrix **Z** came from the set of actual reactions that were removed from the matrix $S_{I}$. On the other hand, for the determination of the FP and TN rates, we repeated the above tests but using a matrix **Z** containing a distinct set of linearly independent reactions of the same size as the set of the omitted reactions. While the arrangement for determining the FP and TN rates does not reflect the null hypotheses in the F- and LM tests, it reflects better the typical scenario in practice (where the true model is unknown). The rates of TP, FN, FP, and TN were computed from 1000 repeated runs of the above tests. Finally, in all of these tests, we ensured that the rank condition for the OLS estimation was always satisfied.

## 3. Results

### 3.1. Case Study I: Specification Bias

In this case study, we considered the CHO metabolic model in Figure 1 with the measured exchange flux values and standard deviations reported previously [16]. We first employed the GLS regression to obtain the estimate of $v_{I}$, denoted by ${\hat{v}}_{I,GLS}$ (see Supplementary Table S2). Figure 1 further depicts the flux distribution according to ${\hat{v}}_{I,GLS}$. Below, we evaluated the impact of omitting a single reaction from the CHO metabolic network in terms of the bias in the estimated flux values and the significance of the linear regression. We computed the specification bias using Equation (15) and reported the bias in relative (percent) values with respect to ${\hat{v}}_{I,GLS}$. Meanwhile, we employed ANOVA (analysis of variance) to establish the statistical significance of the linear regression [29]. 

Table 1 gives the minimum, median, mean, and maximum absolute specification bias for the omission of single reactions, one at a time, from the stoichiometric matrix $S_{I}$. Here, we only removed reactions that would not create an orphan species, i.e, a species that does not participate in any reaction. For each reaction removal, we also generated 10,000 vectors of in silico data of $y=S_{I}v_{I}$ using the full $S_{I}$ matrix and contaminated the data with independent Gaussian random noise with the variance-covariance matrix constructed from the reported standard deviations [16]. For each data vector, we evaluated the significance of regression by ANOVA using the reduced $S_{I}$ matrix, i.e., the matrix $S_{I}$ with a missing column (reaction). The averages of the *p* values from the ANOVA are given in Table 1. Here, we took *p* value of 0.05 as the threshold to reject the GLS regression; any *p* values higher than the threshold indicate a poor regression outcome. 

The individual removal of roughly 3/4 of the reactions (26 out of 36 reactions) still produced a significant regression with *p* < 0.05. On average, the median, mean, and maximum specification biases in the flux estimates were higher for the removal of reactions that caused a poor regression (*p* > 0.05). The two highest *p* values expectedly came from the removal of reactions with the two largest fluxes, and each expectedly had large specification biases. There were nevertheless exceptions where a poor regression resulted from removing a reaction with a moderately low flux value (e.g., reactions 21 and 22). On the other hand, many of the cases with a significant regression (*p* < 0.05) were associated with high maximum specification biases. In fact, several of the cases among the lowest *p* values (i.e., the most significant regression) had a mean bias of >30% and a maximum bias of above 800%. Equally important, the removal of several reactions with a low flux magnitude led to large mean and maximum flux biases (mean bias >150%), as highlighted in Table 1 and by thin red arrows in Figure 1. Therefore, while a poor regression generally points to a model misspecification problem or a violation of the assumption of measurement noise, a statistically significant regression does not guarantee a small specification bias in the flux estimates. In addition, removing reactions with a low flux magnitude can cause disproportionately large specification biases in the flux estimate. These observations clearly motivate the use of a more systematic assessment of the model misspecification issue in the overdetermined MFA.

### 3.2. Case Study II: Stoichiometric Model Misspecification Tests

We evaluated the ability of the Ramsey RESET test, F-test, and LM test to detect the issue of stoichiometric matrix misspecification in the overdetermined MFA, particularly the existence of omitted or missing reactions from **S_I_**. As outlined in Materials and Methods, we determined the rates of TP, TN, FP, and FN using randomly generated pairs of data $y=-S_{E}v_{E}$ and stoichiometric matrices **S_I_** with missing reactions. For the F- and LM tests, we used the information on the actual missing reactions, as well as a distinct set of reactions, as the design matrix of the missing variables **Z**. The results using the baseline MFA problems with 100 metabolites (***m***), 60 unknown internal reactions ($n_{v_{I}}$), 50 measured exchange reactions ($n_{v_{E}}$), and two, five, or 10 missing reactions ($n_{v_{O}}$) for different noise levels (1 to 20% CoV), are summarized in Table 2. Note that by definition, the TP and FN rates sum to 1 and so do the FP and TN rates. 

In general, the results in Table 2 showed that the F-test consistently outperformed the RESET and LM tests and was able to provide high TP rates at moderately low FP rates across all noise levels and different numbers of missing reactions. The results of further evaluations of the F-test performance for metabolic networks of different sizes (*m* = 50 and 200 metabolites and *n* = 55 and 220 reactions, respectively) in Table 3 confirmed the robust performance of the F-test. In general, for the F-test, as the level of measurement noise increased (higher CoV), the TP rates expectedly dropped. We also observed that the smaller the number of missing reactions, the poorer were the TP rates of the F-test. This trend was also expected since, with fewer missing reactions, the reduced **S_I_** was closer to the true system and could more accurately capture the flux balance. Therefore, for the F-test to correctly detect a misspecification of **S_I_**, the missing reactions would need to cause a significant deterioration in the data fitting, a scenario that was less likely to occur as the number of missing reactions became lower. Meanwhile, the FP rates were not a strong function of the noise level. The FP rates improved with a lower number of missing reactions, albeit only slightly. 

With larger networks, detecting the same number of missing reactions using the F-test became more difficult, as expected. At the largest network size (*m* = 200), the rate of correctly detecting a misspecification with two missing reactions was slightly lower than 60%. Fortunately, the TP rates for detecting five or more missing reactions were still high (>88%), and the FP rates depended weakly on the size of the networks and the number of missing reactions and remained relatively low, between 10% to 15%, in most of the cases in our study (see also Supplementary Table S3). 

On the other hand, the RESET test performed very poorly in this case study, in which the FP rates were consistently higher than the TP rates. The general trends observed for the F-test also did not apply to the RESET test. We noted that the RESET test is derived based on the assumption that data error has a constant variance (i.e., the data noise is homoscedastic) [20]. Since the data noise in this case study has a standard deviation that scales linearly with the mean flux value, this assumption was violated. Upon repeating the RESET test using homoscedastic in silico flux data, the RESET test performed much better, with much lower FP rates (see Supplementary Table S4). In addition, the trends of lower TP rates with increasing noise levels and with fewer missing reactions were applicable to the RESET test results when the data noise was homoscedastic. 

The LM test performed better than the RESET test but produced lower TP rates than the F-test, particularly at the highest number of missing reactions ($n_{v_{O}}=10$). The LM test can handle heteroscedastic data through the use of heteroscedasticity-consistent (HC) standard errors in the matrix $\hat{\Omega }$. The results in Table 2 showed that, like the F-test, the TP rates of the LM test decreased with increasing noise levels. Also, as with the F-test, the TP rates of the LM test increased upon increasing the number of missing reactions from two to five, but, upon increasing the number of missing reactions further, the TP rates of the LM test decreased. With an increasing number of missing reactions, the magnitude of the residuals from the OLS estimation using the misspecified model, and therefore the diagonal elements of the HC matrix $\hat{\Omega }$, would become larger. Since the rejection rates of the null hypothesis decreased with larger $\hat{\Omega }$, the FP rates tended to decrease with more missing reactions. For the same reason, the TP rates of the LM test dropped at the highest number of missing reactions.

Considering its robust performance in this case study, we therefore recommend the F-test for detecting model misspecification in the overdetermined MFA. The F-test requires the stoichiometry of the candidate missing reactions as an input. With the extensive knowledge on metabolic reactions available in the literature and in online public databases, such a requirement may not be overly limiting. 

### 3.3. Case Study III: Resolving Model Misspecification

In the last case study, we evaluated the performance of the proposed iterative procedure to resolve stoichiometric matrix misspecifications in the overdetermined MFA (see Materials and Methods). Here, we returned to the flux analysis of the CHO metabolic network in Figure 1. For the performance assessment, we created 100 different stoichiometric matrices **S_I,true_** by randomly removing a number *n_extra_* of columns from the stoichiometric matrix **S_I_** of the CHO model. For each **S_I,true_**, we generated an artificial data vector $y=S_{I,true}v_{I,true}$ using the GLS flux estimate (see Supplementary Table S2) and contaminated the data vector with independent Gaussian noise with zero mean and the variance-covariance matrix, as in Case Study I. The data generation procedure was repeated 100 times. For each data vector, we then created a reduced matrix **S_I,red_** by randomly removing a number *n_omit_* of reactions from **S_I,true_**. The reactions removed in the creation of **S_I,true_** and **S_I,red_** were subsequently combined in the matrix **S_A_**. In other words, the set of candidate missing reactions **S_A_** had equal fractions of the actual omitted reactions and the extra reactions that were not used in the in silico data generation. Finally, we applied the strategy for resolving model misspecification to each data vector using the matrix **S_I,red_** as the reduced stoichiometric matrix and the matrix **S_A_** as the candidate missing reaction matrix. The strategy was implemented using two settings: (1) *k =* 1 and (2) *k* = 1 followed by *k* = 2. 

Table 4 gives the number of reactions in **S_A_** that were not positively identified by the iterative procedure to be included in the stoichiometric matrix. As an indication of good performance, the number of omitted reactions (extra reactions) that remained should be low (high). The results in Table 4 demonstrated that the proposed procedure using *k* = 1 was able to correctly detect and incorporate almost all of the omitted reactions, while keeping the incorrect inclusion of extra reactions low. As expected, performing an additional run with *k* = 2 after finishing the procedure with *k* = 1 led to a higher incorporation rate of the omitted reactions, but such a strategy came at the cost of a higher rate of incorrect addition of the extra reactions. Due to the small size of the CHO model and the number of missing reactions considered, a higher *k* (e.g., *k* = 2) led to the incorporation of all omitted and extra reactions (see Supplementary Table S5). Considering the trade-off above, we thus recommend using a simple implementation with *k =* 1 to resolve the issue of stoichiometric matrix misspecifications in the overdetermined MFA.

## 4. Discussion

Metabolic flux analysis is an indispensable tool for elucidating and understanding the metabolic phenotype of cells, with numerous applications in biotechnology and biomedical fields. The core of the MFA is the stoichiometric model of the metabolic network, which, under pseudo-steady state assumption, enforces a constraint on the distribution of the metabolic flux values. The power of MFA methods stems from their ability to provide estimates of the intracellular metabolic fluxes from measurements of extracellular species concentrations or ^13^C isotopic labeling experiments, using only the stoichiometry on the metabolic reactions. However, because of its reliance on the stoichiometric model, the accuracy of the flux predictions should therefore depend sensitively on the veracity of the stoichiometric matrix in the MFA. Despite its obvious importance, the impact, detection, and rectification of stoichiometric matrix misspecifications have not received much attention in the past. We aimed to fill this gap for the overdetermined MFA, for which the flux estimation problem constitutes an overdetermined linear regression problem. 

Statistical analysis of linear least square regression has provided numerous tools for assessing, among other things, the goodness of fit, gross measurement errors, accuracy of flux estimate, and error propagation in the overdetermined MFA (as presented in Introduction). In this work, we adapted statistical analysis and tests for model misspecifications in linear least square regressions. To the best of our knowledge, such analysis and tests have not yet been applied to the flux estimation problem of the overdetermined MFA. Here, we first derived a simple formula to evaluate the specification bias in the flux estimate due to missing reactions in the stoichiometric model. Using a stoichiometric model of the CHO metabolism, we showed that the significance of the regression is not a sufficient indicator of a low flux bias. In particular, the omission of reaction(s) that results in a high bias (error) in the flux estimate could still produce a statistically acceptable regression. Furthermore, we demonstrated that the removal of reactions with a low flux value could also cause disproportionately large specification biases. In practice, the significance of the regression and the prior information on the magnitude of reaction fluxes are often used for the curation of a reduced order stoichiometric model for the overdetermined MFA. Our findings from the first case study clearly motivated computing the potential flux specification bias during the removal of reaction(s). 

Among the statistical tests that we evaluated for detecting stoichiometric matrix misspecifications, the results from random metabolic networks clearly demonstrated the superiority of the F-test. The F-test could provide high TP rates and low FP rates for detecting missing reactions for most problem sizes (except when there were only very few missing reactions). Based on these findings, we proposed an iterative procedure using the F-test to detect and correct missing reactions. In each iteration, we used the F-test to identify a combination of *k* reactions from the set of candidate missing reactions that would (statistically) significantly improve the linear regression. The combinations that passed a certain *p* value threshold are then incorporated in the stoichiometric model. The application of this iterative procedure to the CHO stoichiometric model indicated that the simple implementation using *k* = 1 gave the most robust performance. The computational cost of performing the iterative procedure scales linearly with the number of candidate reactions when using *k* = 1 or combinatorically when using *k* > 1. Fortunately, since the F-test involves only computationally efficient matrix inversions and matrix-vector multiplications, each iteration of this procedure takes little time to finish. For example, each iteration in Case Study III, with eight missing reactions, using *k* = 1 finished in roughly $4\times 10^{-3}$ seconds on a standalone workstation (MATLAB R2016b, 3.2 GHz Intel Core i5, 16GB memory). The MATLAB codes for the case studies are provided as a supplementary material. 

Finally, there exist several obvious limitations in the statistical tests evaluated in this work. First, these tests rely on the assumption of normality for the noise. The validity of this assumption could be checked by standard normality tests such as the Lilliefors or Shapiro-Wilk tests [30] or using a normal probability plot. When the number of measurements is sufficiently large (>30 for non-skewed noise), the normality assumption is typically reasonable thanks to the central limit theorem. Another limitation of the tests is the imposition on the rank of the matrices, i.e., the set of reaction stoichiometry produces a stoichiometric matrix with a full column rank. In other words, the reactions in the model are non-redundant, and the stoichiometry of the reactions is linearly independent. Note that this rank condition is a requirement for finding a solution to the linear least square regression problem of the overdetermined MFA. While detailed metabolic network models typically have redundant reactions, the metabolic models used in the overdetermined MFA are curated to satisfy the rank condition above; for example, by lumping of reactions and species belonging to a pathway together [31]. Thus, a ‘reaction’ in the overdetermined MFA often represents the overall stoichiometry of multiple reactions. Within the scope of the overdetermined MFA, the F-test also requires that the set of candidate missing reactions have linearly independent stoichiometry with respect to the misspecified model. The candidate reactions again may not represent elementary reactions but could come from the combinations of several reactions to ensure the satisfaction of the rank condition above. 

## 5. Conclusions

In this work, we addressed the misspecification of the stoichiometric matrix in the overdetermined MFA, particularly the omission of reactions. For this purpose, we adapted statistical analysis and tools from linear least square regression to quantify, detect, and resolve the issue of missing reactions. In particular, we derived a simple formula to evaluate the flux bias caused by missing reactions in the overdetermined MFA. We further assessed the performance of several model misspecification tests, namely Ramsey’s RESET test, the F-test, and the Lagrange multiplier test, in detecting missing reactions for overdetermined stoichiometric models. Finally, we proposed an iterative procedure for resolving the issue of missing reactions based on applying the F-test to the candidate missing reactions one at a time and incorporating reactions that pass the significance test. The application of these techniques to the CHO metabolic model and random metabolic networks provided several important conclusions. First of all, the significance of the regression, a common metric for assessing the data-model consistency in the overdetermined MFA, does not guarantee a low bias in the flux estimates. In addition, a high flux bias could result from the removal of a reaction with a low flux magnitude that would be typically deemed unimportant in the construction of the stoichiometric matrix. Therefore, the potential flux bias due to any removal of reaction(s) should be computed during the construction of the stoichiometric model. When the stoichiometry of the candidate missing reactions is known, the F-test provides a robust means, with a high TP rate (nearly 100% for many cases) and a relatively low FP rate (<15%), to detect model misspecifications in the overdetermined MFA. Upon a positive detection of model misspecification in the overdetermined MFA, the proposed iterative procedure in this study gives an effective and robust systematic approach to resolve this issue. 

## Figures and Tables

**Table 1:** Case Study I: Specification bias in the Chinese hamster ovary (CHO) model.

Reaction ^a^	${\hat{v}}_{I,GLS}$	*p* Value ^c^	Absolute Specification Bias (%) ^d^			
	$\left(\frac{\mathrm{nmol}}{10^{6}\;\mathrm{cells}\;h}\right)$		Min	Median	Mean	Max
25	−0.02	0.00 ± 0.00	0.00	0.41	2.73	54.1
19	0.03	0.00 ± 0.00	0.00	0.39	2.48	48.8
10	−1.46	0.00 ± 0.00	0.00	0.15	1.96	11.6
45	−0.21	0.00 ± 0.00	0.00	2.04	18.3	269
17	−0.21	0.00 ± 0.00	0.00	2.11	19.0	280
31	−0.24	0.00 ± 0.00	0.00	2.83	24.9	361
27	0.34	0.00 ± 0.00	0.00	2.12	15.6	229
14	12.50	0.00 ± 0.00	0.00	1.31	33.3	855
9	12.50	0.00 ± 0.00	0.00	1.31	33.3	855
46	15.04	0.00 ± 0.00	0.00	0.88	38.1	1020
8	15.04	0.00 ± 0.00	0.00	0.88	38.1	1020
37	0.27	0.00 ± 0.00	0.00	5.86	54.1	753
12	17.42	0.00 ± 0.00	0.02	1.28	43.1	1190
11	17.84	0.00 ± 0.00	0.02	1.09	44.0	1220
13	18.06	0.00 ± 0.00	0.02	1.66	46.7	1230
30	−0.27	0.00 ± 0.00	0.00	6.87	63.8	889
24	−0.38	0.00 ± 0.00	0.00	6.67	60.9	860
26	0.27	0.00 ± 0.00	0.00	4.19	36.1	509
35	0.13	0.00 ± 0.00	0.00	3.12	28.8	399
33	0.22	0.00 ± 0.00	0.00	11.7	124	2060
32 ^b^	−1.18	0.01 ± 0.01	0.00	21.0	196	2770
29 ^b^	0.99	0.01 ± 0.01	0.00	13.9	170	3840
34 ^b^	0.47	0.01 ± 0.01	0.00	16.2	152	2110
36 ^b^	0.87	0.02 ± 0.01	0.00	23.1	217	3020
23 ^b^	−1.34	0.02 ± 0.01	0.00	17.1	177	2470
28 ^b^	1.03	0.02 ± 0.01	0.00	17.6	158	2220
15	12.31	0.05 ± 0.02	0.00	14.3	121	2210
4	1.24	0.05 ± 0.02	0.00	5.25	65.1	2420
21	−6.81	0.13 ± 0.03	0.00	53.9	475	6980
16	19.26	0.15 ± 0.03	0.00	61.4	573	8100
18	−21.53	0.20 ± 0.04	0.00	61.6	632	8830
43	19.52	0.46 ± 0.04	0.00	74.0	477	8050
22	7.24	0.47 ± 0.04	0.00	121	988	14,700
7	19.63	0.52 ± 0.04	0.00	21.8	114	2360
2	157.77	0.97 ± 0.01	0.00	1.28	803	21,600
3	315.55	0.97 ± 0.01	0.00	1.28	803	21,600

**^a^** The reaction numbers refer to the CHO metabolic network in Figure 1; ^b^ The omission of reactions with a low flux could cause large specification biases in the flux estimate; ^c^ The significance of regression was assessed by ANOVA. The average *p* value (mean ± standard error) was computed for 10,000 GLS regressions using independently generated in silico data; ^d^ The minimum, median, mean, and maximum biases were computed over the remaining reaction fluxes in the model.

**Table 2:** Case Study II: Performance of model misspecification tests (values represent rates).

*m*	$n_{v_{I}}$	$n_{v_{E}}$	$n_{v_{O}}$	CoV	RESET Test (p = 1)				RESET Test (p = 2)				F-Test				LM Test			
					TP	FN	FP	TN	TP	FN	FP	TN	TP	FN	FP	TN	TP	FN	FP	TN
**100**	**60**	**50**	**2**	**0.01**	0.18	0.82	0.56	0.44	0.33	0.67	0.75	0.25	0.86	0.14	0.09	0.91	0.68	0.32	0.11	0.89
				**0.05**	0.28	0.72	0.57	0.43	0.44	0.56	0.78	0.22	0.82	0.19	0.09	0.91	0.67	0.33	0.14	0.86
				**0.1**	0.32	0.69	0.58	0.42	0.51	0.49	0.76	0.24	0.82	0.19	0.10	0.90	0.66	0.34	0.16	0.84
				**0.2**	0.42	0.58	0.56	0.44	0.69	0.31	0.81	0.19	0.71	0.29	0.08	0.92	0.60	0.41	0.18	0.82
			**5**	**0.01**	0.11	0.89	0.57	0.43	0.33	0.67	0.76	0.25	0.99	0.01	0.14	0.87	0.71	0.29	0.07	0.93
				**0.05**	0.12	0.88	0.54	0.46	0.34	0.67	0.73	0.27	0.98	0.02	0.12	0.88	0.73	0.27	0.06	0.94
				**0.1**	0.19	0.81	0.54	0.46	0.41	0.59	0.75	0.25	0.97	0.03	0.13	0.87	0.71	0.29	0.11	0.90
				**0.2**	0.29	0.71	0.55	0.45	0.58	0.42	0.82	0.19	0.93	0.07	0.11	0.89	0.70	0.30	0.12	0.88
			**10**	**0.01**	0.11	0.89	0.57	0.43	0.40	0.60	0.73	0.27	1.00	0.00	0.11	0.89	0.47	0.53	0.00	1.00
				**0.05**	0.13	0.87	0.57	0.43	0.42	0.58	0.76	0.24	1.00	0.00	0.10	0.90	0.48	0.52	0.01	0.99
				**0.1**	0.16	0.84	0.54	0.46	0.47	0.53	0.75	0.26	1.00	0.00	0.13	0.87	0.48	0.52	0.01	0.99
				**0.2**	0.26	0.74	0.57	0.43	0.57	0.43	0.79	0.21	0.99	0.01	0.12	0.88	0.44	0.56	0.01	0.99

**Table 3:** Case Study II: Additional misspecification tests using the F-test (values represent rates).

m	$n_{v_{I}}$	$n_{v_{E}}$	$n_{v_{O}}$	CoV	TP	FN	FP	TN
**50**	**30**	**25**	**2**	0.01	0.86	0.14	0.11	0.89
				0.05	0.82	0.18	0.10	0.90
				0.1	0.75	0.25	0.09	0.91
				0.2	0.69	0.31	0.09	0.91
			**5**	0.01	0.99	0.01	0.10	0.90
				0.05	0.98	0.02	0.10	0.90
				0.1	0.97	0.03	0.10	0.90
				0.2	0.92	0.08	0.11	0.89
			**10**	0.01	1.00	0.00	0.10	0.90
				0.05	1.00	0.00	0.09	0.91
				0.1	1.00	0.00	0.09	0.91
				0.2	0.99	0.02	0.11	0.90
**200**	**120**	**100**	**2**	0.01	0.76	0.24	0.11	0.89
				0.05	0.73	0.27	0.10	0.90
				0.1	0.67	0.33	0.07	0.93
				0.2	0.58	0.42	0.10	0.90
			**5**	0.01	0.97	0.03	0.16	0.84
				0.05	0.95	0.05	0.11	0.89
				0.1	0.94	0.07	0.13	0.87
				0.2	0.88	0.12	0.13	0.88
			**10**	0.01	1.00	0.00	0.15	0.85
				0.05	0.99	0.01	0.16	0.84
				0.1	1.00	0.01	0.13	0.87
				0.2	0.98	0.02	0.15	0.85
			**20**	0.01	1.00	0.00	0.14	0.86
				0.05	1.00	0.00	0.14	0.86
				0.1	1.00	0.00	0.15	0.86
				0.2	1.00	0.00	0.14	0.86

**Table 4:** Case Study III: Iterative procedure for resolving model misspecification in the CHO model.

*k*	*n_extra_*	*n_omit_*	Number of Remaining Reactions ^a^	
			Extra Reactions	Omitted Reactions
1	3	3	2.82 ± 0.38	0.99 ± 0.10
	5	5	4.13 ± 0.63	1.34 ± 0.46
	8	8	5.89 ± 0.83	2.21 ± 0.48
1 then 2	5	5	3.66 ± 0.59	0.97 ± 0.17
	8	8	5.03 ± 0.70	1.00 ± 0.29

**^a^** The number of remaining reactions (mean ± standard error) corresponds to the average over 100 generations of the stoichiometric matrix $S_{I,true}$, of the median number across 100 in silico data simulations.

## Supplementary Materials

The following are available online at http://www.mdpi.com/2306-5354/4/2/48/s1, Figure S1: Coefficient of variation analysis of the fluxes, Table S1: Metabolic reactions and exchange fluxes in the Chinese hamster ovary metabolic model, Table S2: Intracellular flux estimate of the CHO cell culture, Table S3: Case Study II: Other misspecification tests using the F-test, Table S4: Case Study II: Performance of model misspecification tests assuming homoscedastic noise with CoV of the mean flux value (values represent rates), Table S5: Case Study III: Iterative procedure for resolving model misspecification in the CHO model (*k* = 2), and MATLAB codes of the misspecification tests and the flux analysis of the CHO model.
