# Supplementary material for: Assessing and Resolving Model Misspecifications in Metabolic Flux Analysis
Source: Bioengineering (Basel). 2017 May 24;4(2):48. doi: 10.3390/bioengineering4020048 (PMC5590471; doi:10.3390/bioengineering4020048)
Supplement: Supplementary file 1 [file bioengineering-04-00048-s001.zip › bioengineering-187059-final-Supplementary Material/Supplmenatry materials-Assessing and Resolving Model Misspecifications in Metabolic Flux Analysis.pdf]

Supplementary Material for

Assessing and Resolving Model Misspecifications in Metabolic Flux Analysis

Rudiyanto Gunawan and Sandro Hutter

Institute for Chemical and Bioengineering, ETH Zurich, Zurich, Switzerland

Swiss Institute of Bioinformatics, Lausanne, Switzerland

Table S1. Metabolic reactions and exchange fluxes in the Chinese hamster ovary metabolic model (adapted from [16,25]).

#	Formula										
1	Glc	→	G6P								
2	G6P	→	2 DHAP								
3	DHAP	→	Pyr								
4	G6P	→	R5P								
5	Pyr	→	Lac								
6	mAcCoA	→	AcOH								
7	Mal	→	Pyr								
8	mPyr	→	mAcCoA								
9	mAcCoA	+	mOxal	→	mCit						
10	mCit	→	mαKG								
11	mαKG	→	mSucCoA								
12	mSucCoA	→	mSuc								
13	mSuc	→	mMal								
14	mMal	→	mOxal								
15	Oxal	→	Mal								
16	Glu	+	Pyr	→	αKG	+	Ala				
17	mGlu	→	Amm	+	mαKG						
18	Glu	+	Amm	→	Gln						
19	Glu	+	Oxal	→	Asp	+	αKG				
20	Glu	→	Pgl								
21	Ser	→	Pyr	+	Amm						
22	Ser	→	Gly	+	NMFH4						
23	Amm	+	NMFH4	→	Gly						
24	Thr	→	Gly	+	mAcCoA						
25	Cys	+	αKG	→	Glu	+	Pyr				
26	His	→	Glu	+	NMFH4	+	2 Amm				
27	Arg	+	αKG	→	2 Glu						
28	Pro	→	Glu								
29	Asn	→	Amm	+	Asp						
30	Ile	+	αKG	→	mSucCoA	+	Glu	+	mAcCoA		
31	Leu	+	αKG	+	mSucCoA	→	2 mAcCoA	+	mSuc	+	Glu
32	Lys	+	αKG	→	Glu	+	2 mAcCoA	+	Amm		
33	Met	+	Ser	→	mSucCoA	+	Cys	+	Amm	+	NMFH4
34	Phe	→	Tyr								
35	Trp	→	2 mAcCoA	+	Ala	+	Amm				
36	Tyr	+	αKG	+	mSucCoA	→	Glu	+	Mal	+	2 mAcCoA
		+	mSuc								
37	Val	+	αKG	→	mSucCoA	+	Glu				

38	G6P	→	Carb								
39	9 mCit	+	9 Mal	→	OA	+	9 mMal	+	9 Oxal		
40	1.9 Gln	+	1.3 Asp	+	0.5 Gly	+	1.3 NMFH4	+	R5P	→	1.9 Glu
		+	0.8 Mal	+	DNA						
41	2.091 Gln	+	1.194 Asp	+	0.489 Gly	+	0.978 NMFH4	+	R5P	→	2.091 Glu
		+	0.683 Mal	+	RNA						
42	0.095 Ala	+	0.048 Asp	+	0.039 Asn	+	0.063 Arg	+	0.028 Cys	+	0.052 Gln
		+	0.064 Glu	+	0.078 Gly	+	0.022 His	+	0.052 Ile	+	0.088 Leu
		+	0.089 Lys	+	0.02 Met	+	0.021 Phe	+	0.028 Pro	+	0.057 Ser
		+	0.061 Thr	+	0.006 Trp	+	0.02 Tyr	+	0.059 Val	→	Prot
43	mMal	+	αKG	→	mαKG	+	Mal				
44	mMal	+	Cit	→	mCit	+	Mal				
45	Glu	→	mGlu								
46	Pyr	→	mPyr								
47	FoOH	→	NMFH4								
	**Exchange Fluxes**						**Measured**		**Standard deviation**		
		→	Ala				-34.9		1.34		
		→	2 Cys				0.33		0.06		
		→	Gly				-2.39		0.22		
		→	Ser				2.48		0.37		
		→	Thr				1.59		0.17		
		→	Arg				2.38		0.39		
		→	Glu				-0.34		0.41		
		→	Gln				7.08		1.02		
		→	His				0.97		0.15		
		→	Pro				1.93		0.15		
		→	Asp				2.04		0.13		
		→	Asn				2.24		0.20		
		→	Ile				1.41		0.11		
		→	Leu				2.61		0.25		
		→	Lys				1.69		0.12		
		→	Met				0.86		0.05		
		→	Phe				1.15		0.08		
		→	Trp				0.32		0.07		
		→	Tyr				1.05		0.07		
		→	Val				2.18		0.13		
		→	Lac				-291		10.9		
		→	Glc				161		3.93		
		→	Amm				-17.8		4.08		
		→	Carb				-2.14		0.27		
		→	OA				-1.37		0.17		
		→	DNA				-0.31		0.04		
		→	RNA				-0.93		0.11		
		→	Prot				-32.9		4.64		
		→	Ala	+	Gln		18.3		1.96		
		→	FoOH				-7.75		0.19		
		→	AcOH				-1.07		0.06		
		→	Pyr				-2.83		0.17		
		→	Cit				-1.61		0.11		
		→	Pgl				-4.00		0.17		

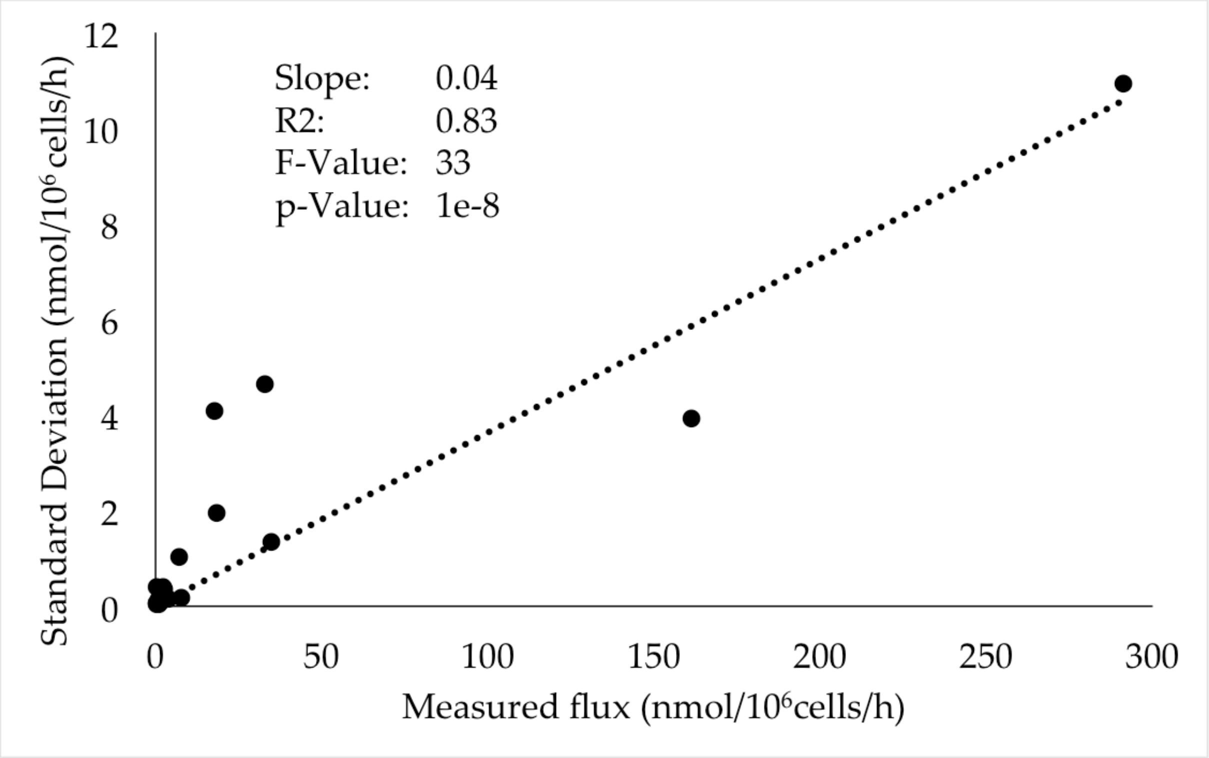

Figure S1 Coefficient of variation analysis of the measured exchange fluxes in CHO cell culture data [16].

Table S2. Intracellular flux estimates (flux distribution) of the CHO cell culture.

Reaction number	$\hat{\mathbf{v}}_{\mathbf{I,GLS}}\left(\frac{\text{nmol}}{\text{1e6cells h}}\right)$
1	161.2
2	157.8
3	315.5
4	1.2
5	291.2
6	1.1
7	19.6
8	15.0
9	12.5
10	-1.5
11	17.8
12	17.4
13	18.1
14	12.5
15	12.3
16	19.3
17	-0.2
18	-21.5
19	0.0
20	4.0
21	-6.8
22	7.2
23	-1.3
24	-0.4
25	0.0
26	0.3
27	0.3
28	1.0
29	1.0
30	-0.3
31	-0.2
32	-1.2
33	0.2
34	0.5
35	0.1
36	0.9
37	0.3
38	2.1
39	1.4
40	0.3
41	0.9
42	32.3
43	19.5
44	-1.6
45	-0.2
46	15.0
47	-7.8

Table S3 Case study II: Other misspecification tests using the F-test (values represent rates)

m	n_{v_I}	n_{v_E}	n_{v_O}	CoV	TP	FN	FP	TN
100	60	45	2	0.01	0.93	0.07	0.10	0.90
				0.05	0.89	0.11	0.11	0.89
				0.1	0.88	0.12	0.10	0.90
				0.2	0.82	0.19	0.09	0.91
			5	0.01	1.00	0.00	0.12	0.88
				0.05	1.00	0.00	0.11	0.89
				0.1	0.99	0.01	0.12	0.88
				0.2	0.99	0.01	0.12	0.89
			10	0.01	1.00	0.00	0.14	0.86
				0.05	1.00	0.00	0.12	0.88
				0.1	1.00	0.00	0.13	0.88
				0.2	0.99	0.01	0.12	0.88
m	n_{v_I}	n_{v_E}	n_{v_O}	CoV	TP	FN	FP	TN
100	60	55	2	0.01	0.97	0.03	0.11	0.90
				0.05	0.95	0.05	0.10	0.90
				0.1	0.91	0.09	0.09	0.91
				0.2	0.83	0.17	0.10	0.90
			5	0.01	1.00	0.00	0.10	0.90
				0.05	1.00	0.00	0.11	0.89
				0.1	0.99	0.01	0.08	0.92
				0.2	0.98	0.02	0.08	0.92
			10	0.01	1.00	0.00	0.13	0.87
				0.05	1.00	0.00	0.11	0.89
				0.1	1.00	0.00	0.11	0.89
				0.2	1.00	0.00	0.11	0.89
m	n_{v_I}	n_{v_E}	n_{v_O}	CoV	TP	FN	FP	TN
100	55	55	2	0.01	0.84	0.16	0.10	0.90
				0.05	0.83	0.17	0.11	0.90
				0.1	0.80	0.20	0.09	0.91
				0.2	0.72	0.28	0.10	0.90
			5	0.01	0.98	0.02	0.13	0.87
				0.05	0.98	0.02	0.14	0.86
				0.1	0.97	0.04	0.13	0.87
				0.2	0.93	0.07	0.11	0.89
			10	0.01	1.00	0.00	0.14	0.86
				0.05	1.00	0.00	0.13	0.87
				0.1	1.00	0.00	0.14	0.86
				0.2	0.99	0.01	0.13	0.87
m	n_{v_I}	n_{v_E}	n_{v_O}	CoV	TP	FN	FP	TN
100	65	45	2	0.01	0.95	0.05	0.10	0.90
				0.05	0.93	0.07	0.11	0.90
				0.1	0.90	0.11	0.10	0.90
				0.2	0.81	0.19	0.08	0.92
			5	0.01	1.00	0.00	0.12	0.88
				0.05	1.00	0.00	0.11	0.90
				0.1	1.00	0.00	0.10	0.90
				0.2	0.98	0.02	0.10	0.90
			10	0.01	1.00	0.00	0.09	0.91
				0.05	1.00	0.00	0.12	0.88

				0.1	1.00	0.00	0.11	0.89
				0.2	1.00	0.00	0.10	0.90

$n_{species}$	n_{vI}	n_{vE}	n_{vOmit}	CoV	TP	FN	FP	TN
50	30	25	2	0.01	0.86	0.14	0.11	0.89
				0.05	0.82	0.18	0.10	0.90
				0.1	0.75	0.25	0.09	0.91
				0.2	0.69	0.31	0.09	0.91
			5	0.01	0.99	0.01	0.10	0.90
				0.05	0.98	0.02	0.10	0.90
				0.1	0.97	0.03	0.10	0.90
				0.2	0.92	0.08	0.11	0.89
			10	0.01	1.00	0.00	0.10	0.90
				0.05	1.00	0.00	0.09	0.91
				0.1	1.00	0.00	0.09	0.91
				0.2	0.99	0.02	0.11	0.90

m	$\boldsymbol{n_{v_I}}$	$\boldsymbol{n_{v_E}}$	$\boldsymbol{n_{v_O}}$	CoV	TP	FN	FP	TN
200	120	100	2	0.01	0.76	0.24	0.11	0.89
				0.05	0.73	0.27	0.10	0.90
				0.1	0.67	0.33	0.07	0.93
				0.2	0.58	0.42	0.10	0.90
			5	0.01	0.97	0.03	0.16	0.84
				0.05	0.95	0.05	0.11	0.89
				0.1	0.94	0.07	0.13	0.87
				0.2	0.88	0.12	0.13	0.88
			10	0.01	1.00	0.00	0.15	0.85
				0.05	0.99	0.01	0.16	0.84
				0.1	1.00	0.01	0.13	0.87
				0.2	0.98	0.02	0.15	0.85
			20	0.01	1.00	0.00	0.14	0.86
				0.05	1.00	0.00	0.14	0.86
				0.1	1.00	0.00	0.15	0.86
				0.2	1.00	0.00	0.14	0.86

Table S4 Case study II: Performance of RESET tests on homoscedastic data (values represent rates).

					RESET test (p = 1)				RESET test (p = 2)			
m	n_{v_I}	n_{v_E}	n_{v_O}	CoV[a]	TP	FN	FP	TN	TP	FN	FP	TN
**100**	**60**	**50**	**2**	**0.01**	0.09	0.91	0.05	0.95	0.21	0.79	0.05	0.95
				**0.05**	0.08	0.92	0.04	0.96	0.19	0.81	0.05	0.95
				**0.1**	0.08	0.92	0.05	0.95	0.20	0.80	0.06	0.94
				**0.2**	0.07	0.93	0.04	0.96	0.17	0.83	0.05	0.95
			**5**	**0.01**	0.09	0.91	0.04	0.96	0.29	0.71	0.05	0.95
				**0.05**	0.10	0.90	0.04	0.96	0.31	0.69	0.04	0.96
				**0.1**	0.11	0.89	0.06	0.94	0.30	0.71	0.05	0.95
				**0.2**	0.10	0.90	0.04	0.96	0.30	0.70	0.04	0.96
			**10**	**0.01**	0.12	0.88	0.06	0.94	0.41	0.59	0.05	0.95
				**0.05**	0.11	0.89	0.05	0.96	0.42	0.58	0.04	0.96
				**0.1**	0.11	0.89	0.05	0.95	0.41	0.59	0.06	0.94
				**0.2**	0.13	0.87	0.06	0.94	0.43	0.57	0.05	0.95

[a]*In silico* were generated in the same manner as in Case Study II, but using independent and identically distributed Gaussian noise with a constant variance (i.e. homoscedastic noise) at the specified CoV.

Table S5 Case study III: Iterative procedure for resolving model misspecification in the CHO model (k = 2).

			Number of remaining reactions[a]	
**k**	n_{extra}	n_{omit}	**Extra reactions**	**Omitted reactions**
2	5	5	0	0
	8	8	0	0

[a]The number of remaining reactions corresponds to the average over 100 generations of the stoichiometric matrix $\mathbf{S}_{\mathbf{I,true}}$, of the median number across 100 in silico data simulations.
